# A systematic review of genetic skeletal disorders reported in Chinese biomedical journals between 1978 and 2012

**DOI:** 10.1186/1750-1172-7-55

**Published:** 2012-08-22

**Authors:** Yazhou Cui, Heng Zhao, Zhenxing Liu, Chao Liu, Jing Luan, Xiaoyan Zhou, Jinxiang Han

**Affiliations:** 1 Shandong Academy of Medical Sciences, Shandong Medical Biotechnological Center, Jinan, 250062, China; 2 Key Laboratory for Biotech Drugs of the Ministry of Health, Key Laboratory for Rare Disease Research of Shandong Province, Jinan, 250062, China

**Keywords:** Rare diseases, Genetic skeletal diseases, China, Bibliographic study

## Abstract

Little information is available on the prevalence, geographic distribution and mutation spectrum of genetic skeletal disorders (GSDs) in China. This study systematically reviewed GSDs as defined in “Nosology and Classification of genetic skeletal disorders (2010 version)” using Chinese biomedical literature published over the past 34 years from 1978 to 2012. In total, 16,099 GSDs have been reported. The most frequently reported disorders were *Marfan syndrome*, *osteogenesis imperfecta*, *fibrous dysplasia*, *mucopolysaccharidosis*, *multiple cartilaginous exostoses*, *neurofibromatosis type 1 (NF1)*, *osteopetrosis*, *achondroplasia, enchondromatosis (Ollier)*, and *osteopoikilosis*, accounting for 76.5% (12,312 cases) of the total cases. Five groups (group 8, 12, 14, 18, 21) defined by “Nosology and Classification of genetic skeletal disorders” have not been reported in the Chinese biomedical literature. Gene mutation testing was performed in only a minor portion of the 16,099 cases of GSDs (187 cases, 1.16%). In total, 37 genes for 41 different GSDs were reported in Chinese biomedical literature, including 43 novel mutations. This review revealed a significant imbalance in rare disease identification in terms of geographic regions and hospital levels, suggesting the need to create a national multi-level network to meet the specific challenge of care for rare diseases in China.

## Introduction

Genetic skeletal disorders (GSDs) arise from disturbances of the complex processes of skeletal development, growth, and homeostasis caused by gene mutations. These disorders represent a challenge in terms of diagnosis and treatment due to their rarity and variety [1,2]. The recently published “Nosology and Classification of Genetic skeletal disorders (2010 version)” listed 456 GSDs that were classified into 40 groups by clinical, radiographic, and molecular criteria; of these, 316 conditions were associated with mutations in 226 different genes [3]. The Nosology not only provides a guideline for the diagnosis of the patients and the recognition of the novel disorders for clinicians, but also is helpful for better understanding the mechanisms of genes, proteins and pathways involved in skeletal biology.

Until now, population-based studies to determine the prevalence of GSDs have been not been performed in China. Most GSDs have been reported in “case reports” in Chinese biomedical literature, but these sources are usually not available to international readers. Therefore, an introduction to the published literature on GSDs in China would enrich our knowledge on the prevalence and molecular characteristics of these rare diseases.

This study systematically reviewed GSDs reported in Chinese biomedical literature published over the past 34 years from January 1978 to January 2012. This study also analyzed the current state and specific challenges in diagnosing and treating rare diseases in China.

## Methods

### Rare diseases covered

This bibliographic study covered a total of 456 GSDs in 40 groups defined in “Nosology and Classification of GSDs (2010 version).” This study has been performed with the approval of the ethics committee of Shandong Academy of Medical Science.

### Selection of database sources

A literature search was conducted using China Biomedical Database (CBM) ( http://sinomed.imicams.ac.cn) and covered sources from January 1978 to January 2012. The CBM is the largest Chinese biomedical bibliographic database [4], and includes a total of 6,840,907 articles from more than 1,600 biomedical journals published in Chinese prior to January 12, 2012.

### Search strategy

The CBM database and public web search engines were first used to search for alternative Chinese terms for the English terms describing each disorder, and then all the terms for the disorder (both in English and Chinese) were used to search for publications in the CBM database. English terms for disorders were included since most Chinese biomedical articles contain an English abstract. The following search algorithm was used: “English disorder terms OR Chinese disorder terms [fulltext]”. For diseases with different subtypes (for example, *Osteogenesis Imperfecta*, types I–V), only the main term (“*Osteogenesis Imperfecta*”) was used in the search, and information on the type was gleaned from the text.

### Inclusion and exclusion criteria

Clinical data and diagnostic information were gleaned from the abstract or full text of the articles searched for in the CBM database. Cases of GSDs with a confirmed diagnosis were included. Detailed clinical, imaging, and laboratory data needed to be described for case reports. Exact diagnostic criteria had to be included for research reports involving multiple cases or families. For each study included, informed consent to publication was obtained from the patient. Patient medical information was carefully compared for series of reports on the same disorders by the same authors or institutions, and redundant cases were excluded.

## Results

According to our criteria 3,208 Chinese reports were qualified for inclusion. A total of 16,099 cases of GSDs in 35 groups of the “Nosology and Classification of Genetic Skeletal Disorders (2010 version)” was reported in the literature. The number of published cases is listed in Table 1. The 10 most frequently reported GSDs were *Marfan syndrome*, *osteogenesis imperfecta*, *fibrous dysplasia*, *mucopolysaccharidosis*, *multiple cartilaginous exostoses*, *neurofibromatosis type 1 (NF1)*, *osteopetrosis*, *achondroplasia*, *enchondromatosis (Ollier)*, and *osteopoikilosis*, accounting for 76.5% of cases (12,312 cases). Five groups (group 8 *TRPV4 group*, group 12 *spondylometaphyseal dysplasias*, group 14 *severe spondylodysplastic dysplasias*, group 18 *bent bones dysplasias*, and group 21 *chondrodysplasia punctata*) described in the Nosology have not been reported yet by Chinese biomedical literature.

**Table 1:** Number of published cases of genetic skeletal diseases in Chinese and Europe biomedical literature listed in alphabetical order of diseases*

**Diseases or group of diseases**	**Number of Cases reported in Chinese biomedical literature**	**Number of published cases in Europe**[8]	**Estimated prevalence in Europe (/100,000)**[8]
Achondroplasia	685		4.5
Acrofacial dysostosis, Nager type	3	90	
alpha-Mannosidosis	1		0.1
Apert syndrome	16		1.25
Asphyxiating thoracic dysplasia	45	150	
Brachydactyly	88	80	
Caffey disease	280	N.A.	
Calcium pyrophosphate deposition disease (familial chondrocalcinosis) type 2	1	N.A.	
Cartilage-hair hypoplasia (CHH; metaphyseal dysplasia,McKusick type)	3	N.A.	
Cherubism	105	N.A.	
Chondrodysplasia punctata	7		0.5
Chondroectodermal dysplasia (Ellis–van Creveld)	11	150	
Cleidocranial dysplasia	260	N.A	
Congenital contractural arachnodactyly	7	N.A.	
Craniofrontonasal syndrome	1	3	
Craniometaphyseal dysplasia	4	70	
Craniostenosis (Craniosynostosis)	302	72	
Crouzon syndrome	161		2
Currarino triad	31		1
de Lange syndrome	7		1.9
Diaphyseal dysplasia Camurati-Engelmann	14	200	
Dysplasia epiphysealis hemimelica (Trevor)	53	N.A.	
Ectrodactyly-ectodermal dysplasia cleft-palate syndrome	5	N.A.	
Ehlers–Danlos syndrome	57		0.2
Enchondromatosis (Ollier)	369	600	
Enchondromatosis with hemangiomata (Maffucci)	80	250	
Endosteal hyperostosis, van Buchem type	8	N.A.	
Familial expansile osteolysis	1	N.A.	
Familial hip dysplasia (Beukes)	45	N.A.	
Familial osteochondritis dissecans	1	N.A.	
Fanconi anemia	107		0.3
Fibrous dysplasia, polyostotic form	982		<50
Frontometaphyseal dysplasia	1	<30	
Frontonasal dysplasia	4	N.A.	
Fucosidosis	1	100	
Fuhrmann syndrome	1	11	
GM1 Gangliosidosis, several forms	7	N.A.	
Grebe dysplasia	2	N.A.	
Greig cephalopolysyndactyly syndrome	2	100	
Hajdu–Cheney syndrome	7	N.A.	
Hallermann–Streiff syndrome	32	<100	
Hanhart syndrome(hypoglossia-hypodactylia)	1	<50	
Holt-Oram syndrome	218		1
Hypertrophic osteoarthropathy	36	N.A.	
Hypochondroplasia	3		3.3
Hypophosphatasia, perinatal lethal and infantile forms	19	N.A.	
Hypophosphatemic rickets	43	<100	
Idiopathic juvenile osteoporosis	1	5	
Immuno-osseous dysplasia (Schimke)	3	50	
Infantile systemic hyalinosis/Juvenile hyaline fibromatosis (ISH/JHF)	3	N.A.	
Klippel-Feil anomaly with laryngeal malformation	318		2
Kniest dysplasia	1	2	
Langer type (Homozygous dyschondrosteosis)	2	N.A.	
Larsen syndrome	9	100	
Lipomembraneous osteodystrophy with leukoencephalopathy (presenile dementia with bone cysts; Nasu–Hakola)	2		0.15
Mandibulo-facial dysostosis(Treacher-Collins, Franceschetti-Klein)	198		6
Marfan syndrome	5064		20
Marshall syndrome	2	63	
Meckel syndrome	35		2.5
Melorheostosis	153	300	
Melorheostosis with osteopoikilosis	6	N.A.	
Mesomelic dysplasia	4	2	
Metaphyseal dysplasia, Jansen type	3	16	
Metaphyseal dysplasia, Schmid type (MCS)	53	N.A.	
Mucolipidosis II (I-cell disease),alpha/beta type	2		0.15
Mucopolysaccharidosis	958		3.56
Multicentric carpal-tarsal osteolysis with and without nephropathy	2	<10	
Multiple cartilaginous exostoses	911		2
Multiple epiphyseal dysplasia	122		5
Multiple sulfatase deficiency	1	50	
Multiple synostoses syndrome	1	20	
Nail-patella syndrome	61		2
Neonatal hyperparathyroidism, severe form	4	N.A.	
Neurofibromatosis type 1 (NF1)	881		25
Oculodentoosseous dysplasia	8	243	
Omodysplasia	1	30	
Oral-facial-digital syndrome	15		1.2
Osteoectasia with hyperphosphatasia (juvenile Paget disease)	4	50	
Osteogenesis imperfecta	1314		6.5
Osteopetrosis	810		1.75
Osteopoikilosis	338	300	
Pachydermoperiostosis (hypertrophic osteoarthropathy,primary, autosomal dominant)	25	204	
Pallister-Hall syndrome	1	100	
Parietal foramina	11		5
Pfeiffer syndrome	4		1
Poland anomaly	27	3	
Preaxial polydactyly	16		25
Progeria, Hutchinson–Gilford type	20		0.005
Progressive osseous heteroplasia	20	N.A.	
Progressive pseudorheumatoid dysplasia (PPRD; SED with progressive arthropathy)	6	N.A.	
Proteus syndrome	22	200	
Proximal symphalangism	15	N.A.	
Pseudoachondroplasia (PSACH)	51		1.6
Pyknodysostosis	14		0.13
Pyle disease	2	<30	
Radio-ulnar synostosis	55	<20	
Saethre–Chotzen syndrome	1		3
Schwartz–Jampel syndrome (myotonic chondrodystrophy)	4	100	
SED tarda, X-linked (SED-XL)	156		0.55
SED, Wolcott–Rallison type	1	<60	
Short rib-polydactyly syndrome	57	N.A.	
Shprintzen-Goldberg syndrome	1	<50	
Sotos syndrome	38		7
Split hand-foot malformation	25		1.1
Spondylocostal dysostosis	1	4	
Spondyloepiphyseal dysplasia congenita (SEDC)	50		0.34
Spondylometaphyseal dysplasia	6		1
Spondylometaphyseal dysplasia, Kozlowski type	2		0.1
Sterile multifocal osteomyelitis,periostitis, and pustulosis (CINCA/NOMID-like)	1	N.A.	
Stickler syndrome	7		13.5
Syndactyly type 5 (HOXD13)	7	N.A.	
Thanatophoric dysplasia	28		3.5
Thrombocytopenia-absent radius	1	N.A.	
Tibial hemimelia	2		0.1
Trichorhinophalangeal dysplasia	15	>100	

*: Different types belong to one diseases have been combined as one item when the typing information was not provided in literature.

**Figure 1:**
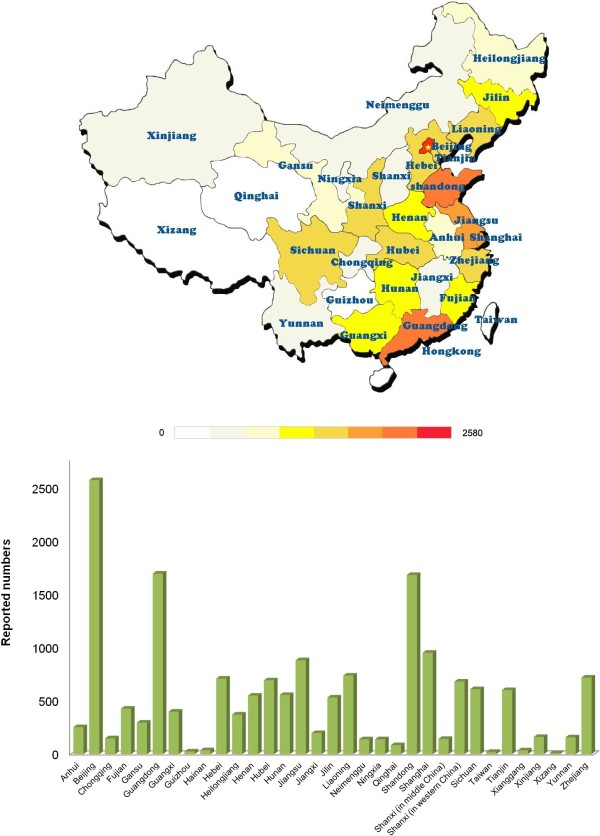
**Geographic distribution of reported cases of genetic skeletal disorders (GSDs) in Chinese biomedical literature.** The number of cases reported varied geographically, and focused in the East and South of China. Beijing, Guangdong, Shandong, Shanghai and Jiangsu ranked among the top 5 provinces or province-level municipalities where GSDs were reported.

The geographic distribution of cases is shown in Figure 1. GSDs have been reported in all of China’s provinces and province-level municipalities. However, the number of cases varied geographically. More patients were reported in the East and South of China, which have a higher population density and better medical services than other areas. Beijing, Guangdong, Shandong, Shanghai and Jiangsu ranked among the top 5 provinces or province-level municipalities where disorders were reported.

**Figure 2:**
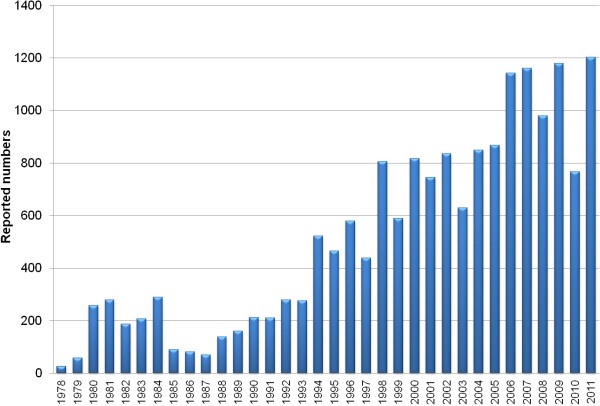
**Case number of genetic skeletal disorders reported in Chinese biomedical literatures from 1978 to 2011.** The number of GSDs reported each year in the CBM database increased gradually since 1978 and rapidly increased starting in 1994. 1,057 cases were reported annually in recent 5 years.

**Figure 3:**
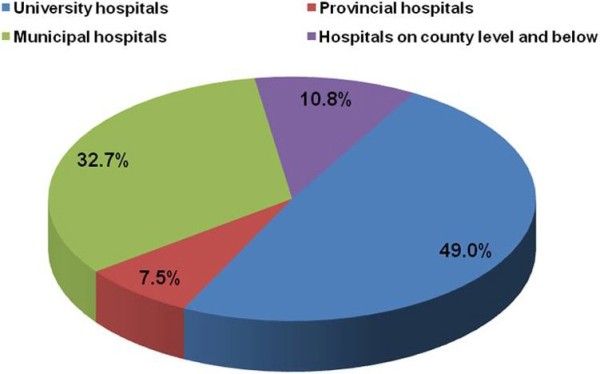
**Hospital distribution of reported cases of genetic skeletal disorders in Chinese biomedical literatures.** Most of the GSDs were diagnosed in hospitals in cities including university hospitals (49.0%), provincial hospitals (10.8%), and municipal hospital (32.7%). Only 7.5% of GSD cases were reported by hospitals on country level and below, which account for about 70% of the medical resources in China.

As shown in Figure 2, the number of patients with GSDs reported each year in the CBM database increased gradually since 1978 and rapidly increased starting in 1994. Most GSD cases were reported by pediatricians, radiologists, and orthopedists. 49.0% of the cases were diagnosed at a university hospital,10.8% were diagnosed at a provincial hospital, 32.7% were diagnosed at a municipal hospital, and the remainder (7.5%) was diagnosed at hospitals on country level or even from smaller communities. (Figure 3).

**Figure 4:**
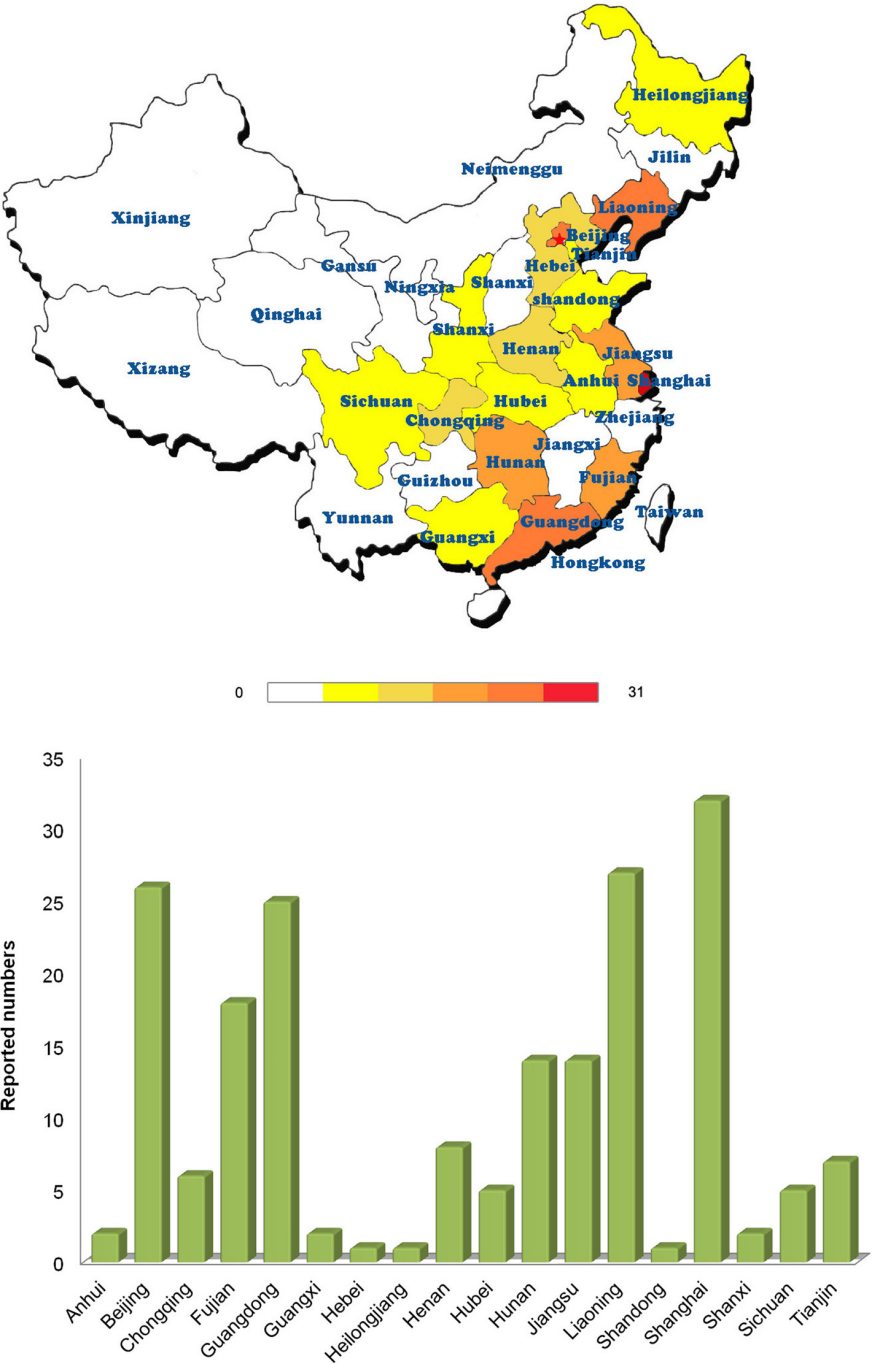
**Geographic distribution of reported genetic skeletal disorders with gene mutation testing in Chinese biomedical literature.** Genetic testing for GSDs was only performed at university hospitals in a few areas, far less than the regions where GSDs were reported.

Gene mutations were evaluated in 187 cases or families out of 16,099 total reported cases, accounting for only a minor portion (1.16%). As shown in Table 2, a total of 37 genes for 41 different GSDs were reported, including 43 novel mutations that have not been reported before. The EXT1 and EXT2 genes (30 cases) for *multiple cartilaginous exostoses*, the FBN1 gene for *Marfan syndrome* (24 cases), and the FGFR3 gene for *achondroplasia* (22 cases) were most frequently reported in Chinese biomedical literature from the CBM database. Compared to the reported geographic distribution of GSDs (Figure 2), genetic testing was only performed at university hospitals in a few areas (Figure 4). Affiliated hospitals of Shanghai Jiaotong University, Chinese Academy Of Medical Science & Peking Union Medical College, Zhongshan University, Central South University, and Peking University rank the top 5 University hospitals which performed most gene testing of GSDs.

**Table 2:** Gene mutation of genetic skeletal disorders published in Chinese biomedical literature from 1978 to 2012*

**Gene**	**Name of disorder**	**MIM No.**	No. of case reported*	**Mutation**	**Location**	**Type**	**Novel**
ACVR1	Fibrodysplasia ossificans progressiva (FOP)	135100	2	c.617 G > A (p.R206H)	exon 4	missense	
			1	c.1067 G > A (p.G356D)	exon 7	missense	
ALPL	Hypophosphatasia, infantile forms	241500	1	c.18delA and c.G407C (p.V7Yfs18X and p.R136P)	exon 2 and 5	nonsense and missense	yes
ALPL	Hypophosphatasia, adult form	146300	1	c.1366 G > A (p.G456R)	exon 12	missense	
			1	c.1581C > G (p.P446G)	exon 12	missense	yes
			1	c.583 G > A (p.R136H)	exon 5	missense	
CLCN5	Dent’s disease	300554	1	p.L594fsX595	exon 10	nonsense	yes
			1	p.R637X	exon 10	nonsense	
			1	p.R467X	exon 9	nonsense	
			1	p.IVS4-2A > G	exon 4	splicing	yes
			1	c.1022C > T (p.S244L)	exon 7	missense	
			1	c.1805 T > G (p.V505G)	exon 9	missense	yes
CLCN7	Osteopetrosis, late-onset form type 2 (OPTA2)	166600	1	c.C856T (p.R286W)	exon 10	missense	
COL1A2	Osteogenesis imperfecta		1	c.A3350G (p.Y1117C)	exon 49	missense	
			1	c.G3305C (p.G1102A)	exon 49	missense	
COL1A1	Osteogenesis imperfecta	166220	1	c.1678 G > A (p.G560S)	exon 25	missense	
		166200	1	p.Gly632x	exon 28	nonsense	yes
		166200	1	p.D1441H	exon 52	missense	yes
		166200	1	c.1875 + 1 G > A (IVS 27 + 1 G > A)	intron 27	splicing	yes
		166200	1	IVS8-2A > G	intron 8	splicing	yes
			1	c.2461 G > A (p.G821S)	exon 36	missense	
			1	c.3470 G > A (p.G1157D)		missense	yes
COL2A1	Spondyloepiphyseal dysplasia congenita (SEDC)	183900	1	c.1510 G > A (p.G504S)		missense	
			1	c.2401 G > A (p.G801S)		missense	
EFNB1	Craniofrontonasal Syndrome	304110	1	c.161C > G (p.P54R)	exon 2	missense	yes
COMP	Pseudoachondroplasia	177170	1	c.815 T > C (p.L272P)	exon 8	missense	
EIF2AK3	SED, Wolcott–Rallison type	226980	1	c.1408-1409insT and c.1596 T > A (p.S470FfsX7 and p.C532X)	exon 8 and 9	nonsense	
EXT1	Multiple cartilaginous exostoses 1	133700	1	c.1564-7delC	exon 7	frameshift	yes
			1	I8 + 2 T > G	intron 8	splicing	yes
			1	c.651_664delinsTTT (p.K218fsX220)	exon 1	nonsense	
			1	c.680delG (p.R227fs)	exon 1	frameshift	
			1	c.1182delG (p.Arg394SerfsX9)	exon 4	nonsense	yes
			1	c.1108 G > T (p.E370X)	exon 3	nonsense	yes
			1	c.335delA (p.Asn112ThrfsX24)	exon 1	nonsense	yes
			1	c.361C > T (p.Q121X)	exon 1	nonsense	yes
			1	c.1879_1881delCAC (p.His627del)	exon 9	In frame deletion	yes
			1	c.651_664delinsTTT (p.K218fsX220)	exon 1	frameshift	
			1	1633-26(C > A)	intron 7	splicing	
			1	c.2120delT	exon 6	frameshift	
			1	c.811 T > C (p.Y271H)	exon 1	missense	yes
EXT2	Multiple cartilaginous exostoses 2	133701	1	c.668 G > C (p.Arg223Pro)	exon 2	missense	
			1	c.950delT (p.Phe317SerfsX15)	exon 6	nonsense	yes
			3	c.1016 G > A (p.Cys339Tyr)	exon 6	missense	
			1	c.398 T > G (p.L133R)	exon 2	missense	
			1	c.751C > T (p.Q251X)	exon 5	missense	
			1	c.544C > T (p.R182X)	exon 3	missense	
			1	c.536 G > A (p.Arg179Lys)	exon 2	missense	
			1	c.1006C > T (p.G1n336X)	exon 6	nonsense	yes
			1	IVS2 + 1 G > A	intron 2	splicing	
			1	IVS7 + 1 G > T	intron 7	splicing	
			1	c.789-796delTGTT	exon 5	frameshift	yes
			1	c.637 G > A	exon 4	nonsense	
			1	c.313A > T (p.Lys105X)	exon 2	nonsense	
			1	319insGT	exon 2	frameshift	
			1	536 + 1 G > A (IVS2 + 1 G > A)	intron 2	splicing	
FBN1	Marfan syndrome	154700	1	c.3463 G > A (p.Asp1155Asn)	exon 27	missense	yes
			1	c.5015 G > C (p.C1672S)	exon 40	missense	
			3	c.5309 G > A (p.C1770Y)	exon 43	missense	
			2	c.7241 G > A (p.R2414Q)	exon 58	missense	
			2	c.7769 G > A (p.C2590Y)	exon 62	missense	
			2	c.2261A > G (p.Y754C)	exon 18	missense	
			1	c.[6862_6871delGGCTGTGTAG;6871 + 1_6871 + 11delGTAAGAGGATC] (p.Gly2288MetfsX109)	exon 55	nonsense	yes
			1	c.2462 G > A (p.Cys821Tyr)	exon 20	missense	yes
			1	c.5015 G > C (p.C1672S)	exon 40	missense	
			1	c.3295 G > T (p.E1099X)	exon 26	nonsense	
			2	c.4307insTCGT (p.G1441X)	exon 34	nonsense	yes
			1	c.4621C > T (p.R1541X)	exon 37	nonsense	
			1	c.8080C > T (p.A2694X)	exon 64	nonsense	
			2	IVS29 + 4A > T	intron 29	splicing	
			1	IVS50 + 1 G > A	intron 50	splicing	
			1	c.3069 G > T (p.Lys1023Asn)	exon 24	missense	yes
			1	c.3243-3256delGCCTCTGCACCCA	exon 25	frameshift	
FGFR1	Pfeiffer syndrome	101600	1	c.755C > G (p.Pro252Arg)	exon 5	missense	
FGFR2	Pfeiffer syndrome	101600	1	IVS8 A > G	exon 8	splicing	
			1	p.Asp321Ala	exon 9	missense	
	Apert syndrome	101200	1	c.934C > G (p.S252W)	exon 7	missense	
	Crouzon syndrome	123500	1	c.833 G > T (p.C278F)	exon 8	missense	
			1	p.Tyr340His	exon 9	missense	
			1	p.Cys342Trp	exon 9	missense	yes
			1	p.Cys342Tyr	exon 9	missense	
			1	p.Ala344Ala	exon 9	missense	
			1	p.Gly338Arg	exon 9	missense	
			1	p.Ala344Gly	exon 9	missense	
			1	p.Gln289Pro	exon 7	missense	
FGFR3	Achondroplasia	100800	21	c.1138 G > A (p.G380R)	exon 10	missense	
			1	p.Ser217Cys	exon 5	missense	
FGFR3	Thanatophoric dysplasia	187600	1	c.742C > T (p.R248C)	exon 7	missense	
GALNS	Mucopolysaccharidosis type 4A	253000	1	c.1567 T > G and c.374C > T	exon 14 and 4	nonsense and missense	yes
GDF5	Multiple synostoses syndrome type 2	186500	2	c.1471 G > A (p.E491K)	exon 2	missense	
GNAS1	Albright hereditary osteodystrophy	103580	1	1-bp (C) deletion at codon 291	exon 11	frameshift	
HLXB9	Currarino triad	176450	2	c.552C > G (p.Tyr184X)		nonsense	
TP63	Split hand-foot malformation, isolated form, type 4 (SHFM4)	605289	1	c.956 G > A (p.R280H)	exon 7	missense	
HOXD13	Synpolydactyly	186000	1	c.32 G > C (p.G11A)	exon 1	missense	
			1	c.64 G > T (p.A22S)	exon 1	missense	yes
			3	9-residue polyalanine expansion	exon 1		
	yes						
			1	8-residue polyalanine expansion	exon 1		
			1	7-residue polyalanine expansion	exon 1		
IDS	Mucopolysaccharidosis type 2	309900	1	c.892C > T (p.Q298X)	exon 7	nonsense	
			1	c.1468delA	exon 9	frameshift	
			1	c.263 G > A (p.Arg88His)	exon 3	missense	
			1	1103_1123del19	exon 8	frameshift	
IHH	Brachydactyly type A1	112500	1	c.G298A (p.D100N)	exon 1	missense	
NF1	Neurofibromatosis type 1	162200	1	c.1009 G > T	exon 7	nonsense	yes
			1	c.3443-3444delCA	exon 20	frameshift	yes
			1	c.4339C > T (p.G1336X)		nonsense	
			1	c.5839C > T (p.R1947X)	exon 31	nonsense	
			1	p.Leu1141Arg	exon 20	missense	
TP63	Ankyloblepharon-ectodermal dysplasia-cleft lip/palate	106260	1	c.838C > T (p.R280C)	exon 7	missense	
	Limb-mammary syndrome (including ADULT syndrome)	603273	1	c.893 G > A (p.R298Q)	exon 8	missense	
PHEX	Hypophosphatemic rickets, X-linked dominant	307800	1	IVS20-1 G > T	intron 20	splicing	
			1	c.1861C > T (p.GIn621X)	exon 18	missense	yes
PTPN11	Baller–Gerold syndrome	218600	1	IVS11-1 G > A and c.3401A > T	intro 11 and exon 10	splicing and nonsense	
ROR2	Brachydactyly type B	113000	1	c.2265C > A (p.Y755X)	exon 9	nonsense	
			1	c.1398-1399insA	exon 9	nonsense	
RUNX2	Cleidocranial dysplasia	119600	1	c.346 T > A (p.W116R)	exon 1	missense	
			1	c.610A > T (p.K204X)	exon 3	nonsense	
			1	c.346 T > A (p.W116R)	exon 1	missense	
			1	c.475 G > C (p.G159R)	exon 2	missense	yes
			1	c.673C > T (p.R225W)	exon 3	missense	
			1	c.1171C > G (p.R391X)	exon 7	nonsense	
			1	c.674 G > A (p.R225Q)	exon 3	missense	
SALL1	Townes–Brocks syndrome (Renal-Ear-Anal-Radial syndrome)	107480	4	c.1792 G > C	exon 2	missense	
SEDL	SED tarda, X-linked (SED-XL)	313400	1	c.218C > T (p.S73L)	exon 4	missense	
			1	c.370-371insA (p.S124fsX127)	exon 6	nonsense	yes
			1	c.218C > T (p.S73L)	exon 4	missense	
			1	c.239A > G (p.H80R)	exon 4	missense	
			1	c.G209A	exon 4	nonsense	
			1	c.262-266delGACAT	exon 5	frameshift	
			1	D109-S123del (p.S124fsX126)	intron 5-exon 6	nonsense	
			1	IVS5-2-1delAG322-332delTTTTCAATGAA	intron 5-exon 6	splicing	yes
			1	IVS2-2A > C	intron 2		
SH3BP2	Cherubism	118400	5	c.1505 G > C (p.Arg415Pro)	exon 9	missense	
			2	c.G1520A (p.Gly420Glu)	exon 9	missense	
SHOX	Dyschondrosteosis	127300	1	c.115 T > G	exon 2		
			1	c.1171-1172insA	exon 3	frameshift	
			1	c.996A > T (p.E102V)	exon 3	missense	
SOX9	Campomelic dysplasia (CD)	114290	1	p.R178L	exon 2	missense	yes
TBX5	Holt-Oram syndrome	142900	1	c.416delC	exon 4	frameshift	
			1	c.145C > A	exon 2	missense	
			1	c.161 T > C	exon 2	missense	
TGFbeta1	Diaphyseal dysplasia Camurati-Engelmann	131300	1	p.R218H	exon 4	missense	
WISP3	Progressive pseudorheumatoid dysplasia	208230	1	c.624-625insA and c.729-735delGAGAAAA	exon 4 and exon 4	frameshift and frameshift	yes
			1	c.624-625insA and c.866-867insA	exon 4 and exon 5	frameshift and frameshift	yes
			1	c.866_867insA and c.866-867insA	exon 5 and exon 5	frameshift and frameshift	yes
			1	c.589 + 2 T > C and c.624dupA	intro 3 and exon 4	splicing and nonsense	yes

*: “Pedigree mutation”, that is an identical mutation has been reported in more than one affected siblings in a family, was counted as one case. Mutation information was extracted from the full text, as its original description, all the novel mutations were claimed in the papers by the authors, and then were confirmed by searching the previous literature and the Human Gene Mutation Database.

## Discussion

As the world’s most densely populated nation, China has the world’s largest number of rare disease groups [5]. In 1984, the concept of rare diseases was introduced in China. Until recently, however, the problem presented by rare diseases has received little attention [6]. Currently, there is no case registration system for most rare diseases, so there is very little documented information on the epidemiology of those diseases in China [7]. China still lacks an official definition and spectrum of rare diseases.

A bibliographic study will help to estimate the prevalence of rare diseases [8]. Most rare diseases have been reported in Chinese biomedical publications. Presently, only 86 Chinese biomedical journals have abstracts in English included in Pubmed [9]. Therefore, most reports on rare diseases in China are unavailable to international readers. To our knowledge, the current study is the first systematic review of the Chinese biomedical literature on rare disease groups.

GSDs are representative for many other groups of rare diseases. The current systematic review found that the number and type of GSDs reported in Chinese biomedical literature increased gradually over the past 30 years. In the last 5 years in particular, there were 1,057 cases reported annually, which is due to the rapid improvement of general healthcare and increasing attention to the medical problems caused by rare diseases in China. Although most genetic skeletal disease groups have been reported in Chinese biomedical literature, but only a small portion of patients were exactly molecularly characterized. For example, 1,314 cases of *osteogenesis imperfecta* were reported in the CBM database but in only 5% the exact type has been determined. This situation might be mainly due to the fact that most of these patients were diagnosed based on clinical and radiographic criteria and because gene mutation testing has been unavailable at most hospitals until now. In only 1% of all cases with GSDs a causative gene mutation was identified. Among these reported mutations, there is a relatively high frequency of novel mutations. These novel variations may also lead to a better understanding of the mutation spectrum and impact of genes associated with GSDs. For example, 5 cases of novel mutations in COL1A1 have been reported in Chinese biomedical literature. Among them, 4 cases belong to glycine single base substitution mutations in the triple-helical region (p.G632x, p.G1157D) and splicing sites (IVS27 + 1 G > A, IVS8-2A > G), which are the most and second common mutation types in COL1A1 gene. Mutations in the C-propeptide coding region have been identified less frequent than other forms of mutation. D1441 is one of a few residues absolutely conserved in this region, a previous study reported a defect in this site (D1441Y) resulting in a lethal variant of osteogenesis imperfecta with features of dense bone diseases [10], however, a novel mutation in this site described in a Chinese family (D1441H) led to only mild osteogenesis imperfecta (type 1), which suggesting mutations in this region show great heterogeneity in clinical outcome.

This systematic review of genetic skeletal diseases also revealed that reporting of rare diseases varies significantly in different regions and medical resources available in China. Cases of rare diseases were more frequently reported in large municipalities such as Beijing and Shanghai instead of areas with a larger population but a relatively lower level of development such as Sichuan and Henan Provinces. Country level hospitals and below are mainly responsible for treating rural residents and represent more than 70% of the medical resources in China [11]. In this review, we found that only 7.5% of the cases of rare diseases were diagnosed by these hospitals, which is significantly lower than that diagnosed by urban hospitals. In actuality, there are also significant disparities in health care between university hospitals and provincial and municipal hospitals. Currently, gene mutation testing for genetic rare diseases in China is done only by university hospitals in several key municipalities.

We further compared the number of GSD cases reported in Chinese biomedical literature with those of published cases or estimated prevalence of these diseases in Europe from bibliographic data issued by Orphanet [8] (Table 1). Generally, the number of GSDs reported in Chinese biomedical literatures is lower than in Europe, but, with some exceptions, the proportions between the different entities are similar. One of these exceptions are the multiple epiphyseal dysplasias, whose frequency in Europe is 5/100,000, while only a total of 122 cases were reported in Chinese biomedical literature in the past 34 years. Although publication bias and genetic differences between Caucasian and Asian people may exist, we think this discrepancy is mainly due to the fact that China is still lagging behind Europe in terms of the medical resources for these rare diseases, especially in the widespread underdeveloped regions and hospitals on basic levels, therefore, many patients with genetic skeletal disorders could not acquire proper and timely diagnosis in China.

Creating a network for rare diseases is an important medical policy that should significantly reduce misdiagnosis and improve the level of treatment. A network for collaboration with national medical resources has been set up in countries and regions such as Europe, North America, and Japan [12,13]. A number of centers offering counseling on rare diseases have been established in major Chinese cities and several provinces, but a national network has yet to be created. Given the fact that there is a huge gap in terms of medical services in different areas and hospital levels of China, a stronger network of diagnosis and treatment including all levels of hospitals across the country should be created to improve healthcare for rare diseases in the future.

## Conclusion

In conclusion, this systematic review summarized the number, geographic and genetic characteristics of GSDs in Chinese biomedical publications. Analyzing number of the diseases revealed an imbalance in the distribution of areas and hospitals diagnosing rare diseases, which suggests that a multi-level network should be created to meet the specific challenge of healthcare for rare diseases in China.

## Competing interests

The authors declare that they have no competing interests.

## Authors’ contributions

JH and YC put forward the idea and designed the key points. YC was responsible for the article writing and data analysis. HZ, ZL and CL were responsible for the data collection. HZ, JL and XZ participated in data analysis. All authors read and approved the final manuscript.
